# Applying a Power and Gender Lens to Understanding Health Care Provider Experience and Behavior: A Multicountry Qualitative Study

**DOI:** 10.9745/GHSP-D-22-00420

**Published:** 2023-11-30

**Authors:** Pooja Sripad, Summer Peterson, Daoudou Idrissou, Martha Kamanga, Abigail Kezembe, Charity Ndwiga, Chantalle Okondo, Anja Noeliarivelo Ranjalahy, Natacha Stevanovic-Fenn, Charlotte E. Warren, Brady Zieman, Sanyukta Mathur

**Affiliations:** a Population Council, Washington DC, USA.; b University of North Carolina, Chapel Hill, NC, USA.; c Country Liaison Associate, Ouagadougou Partnership Coordination Unit, Lome, Togo.; d Kamuzu University of Health Sciences, Lilongwe, Malawi.; e Population Council, Nairobi, Kenya.; f Tandem SARL, Antananarivo, Madagascar.; g Georgetown University, Washington DC, USA.

## Abstract

A limited but growing body of literature shows that health care providers (HCPs) in reproductive, maternal, and newborn health face challenges that affect how they provide services. Our study investigates provider perspectives and behaviors using 4 interrelated power domains—beliefs and perceptions; practices and participation; access to assets; and structures—to explore how these constructs are differentially experienced based on one's gender, position, and function within the health system. We conducted a framework-based secondary analysis of qualitative in-depth interview data gathered with different cadres of HCPs across Kenya, Malawi, Madagascar, and Togo (n=123). We find across countries that power dynamics manifest in and are affected by all 4 domains, with some variation by HCP cadre and gender. At the service interface, HCPs' power derives from the nature and quality of their relationships with clients and the community. Providers' power within working relationships stems from unequal decision-making autonomy among HCP cadres. Limited and sometimes gendered access to remuneration, development opportunities, material resources, supervision quality, and emotional support affect HCPs' power to care for clients effectively. Power manifests variably among community and facility-based providers because of differences in prevailing hierarchical norms in routine and acute settings, community linkages, and type of collaboration required in their work. Our findings suggest that applying power—and secondarily, gender lenses—can elucidate consistencies in how providers perceive, internalize, and react to a range of relational and environmental stressors. The findings also have implications on how to improve the design of social behavior change interventions aimed at better supporting HCPs.

## Abstract (precis)

Applying a power lens to understand provider behavior illuminates how interpersonal, social, and structural relations influence health care providers' power to provide high-quality care.

## Teaser

**Key Findings:**

- Health care providers' (HCPs') power to deliver high-quality care is influenced by perceptions of relationships and interactions with clients, families, peers, and supervisors, as well as interprovider collaboration and community norms.
- HCPs' power is constrained by restrictive or shifting institutional policy and limited access to resources, advancement opportunities, and supportive supervision.
- Interprovider power dynamics suggest that too much HCP power over can sometimes be as consequential as a lack of power to providing quality care.
- Community- and facility-based HCPs' perspectives suggest variable power-enhancing approaches are needed to positively affect a client's experienced quality of care.

**Key Implications:**

- Program stakeholders should consider participatory mechanisms for routine community feedback (positive and negative) to improve the client-provider interaction experiences.
- Facility managers should consider team-building strategies to overcome challenging interprovider dynamics and reinforce collaboration and trust.
- Policymakers and national stakeholders should consider HCPs' power-influencing factors within provider behavior change efforts, including allocations for equitable access to resources and supervision, provider growth opportunities, and guidance for task-sharing.

## INTRODUCTION

Applying power and gender lenses in health systems research is increasingly recognized as a critical area of investigation with implications for social and behavior change (SBC) strategies to enhance quality of health services. These lenses offer insights into how hierarchies in health, social, and economic spheres intersect at health service interactions between health care providers (HCPs) and the clients they serve, often manifesting as positive or negative experiences for clients and providers.^1^^,^^2^ HCPs in reproductive, maternal, and newborn health (RMNH) face environmental, structural, and gender-related challenges in their work, as well as inequitable societal and gender norms, that are internalized and consequently affect provider behaviors and client experience.^3^^–^^7^ Examining HCP perspectives through power and gender lenses is particularly useful to improve understanding of their ability to access resources, navigate normative practices, interpret guidelines and roles, make decisions, and do their work.^8^ Few empirical studies to date have focused on exploring power and gender through provider perspectives in relation to their performance and behavior.^9^ While power and gender underpinned landscape analyses of disrespect and abuse in maternity care provision and experience, prevailing power analyses that apply a range of approaches (case study, discourse, online library actor interface, critical ethnographic, and framework-based analyses) focus on infectious diseases, including childhood illnesses and HIV, as well as health-area agnostic systems areas such as governance/policy implementation, community health services/programs or emergency care systems.^10^^–^^15^

Power, a relational notion, is present between different types of HCPs within a health system and between HCPs and their clients. It can be defined broadly as the relational force that characterizes and influences the capability to make a choice or act in a particular way for oneself and others.^9^^,^^16^ Power often derives from various sources—political, bureaucratic, financial, networks/access, and personal attributes—and may be expressed in distinct ways in health systems.^9^ For HCPs, several types of power may be at play: their power within (internal capability or sense of self-worth and self-knowledge), power to (agency to act in a certain way despite constraints and opposition—e.g., serve a client), power with (collaborating to provide health services), and power over (leveraging resources and challenging authority with greater medical expertise or age).^16^^,^^17^ For example, how a nurse experiences/views power to do her work may vary from how a community health worker (CHW) or a specialist doctor experiences/views hers. Intersecting with power, gender is a social stratifier and construct that elucidates sociocultural definitions of women's and men's roles in a particular society. As health systems sit within and reflect the values of the societies in which they are based, they can reinforce gendered values, power relations, occupational opportunities, and norms in ways that affect relationships during health care interactions.^8^ Although gender-transformative SBC programs have gained traction recently, they have often focused on communities rather than health care settings, where HCP perspectives are integral.

To elucidate how these constructs manifest in an array of health service and geographic settings and inform a more transformative approach to SBC programming, we apply a power and gender lens to the analysis of qualitative data from HCPs in different cadres and countries. We draw on Betron et al.'s mapping of quality care drivers, which uses the U.S. Agency for International Development's gender analysis framework, to map how power dynamics and gender inequities intersect to affect HCPs' provision of care and clients' experience of care.^18^^,^^19^ Although several power frameworks and project-specific primary data-driven quality-of-care improvement strategies exist, the review by Betron et al. topically aligned with our HCP data in the RMNH sphere and was guided by a gender lens that led to a power- and gender-integrated mapping.^9^^,^^20^ The review showed that power and gender pervasively influenced behaviors in social and health systems and could be understood through 4 overlapping domains: (1) beliefs and perceptions;(2) practices and participation; (3) access to assets; and (4) structures—institutions, laws and, policies.^19^

Using a gender analysis framework, we map how power dynamics and gender inequalities intersect to affect HCPs' provision of care and clients' experience of care.

In this article, we use these 4 domains (Figure) to explore manifestations of power and, to a lesser extent, gender from HCPs' perspectives and how these relational dynamics affect provider behaviors with clients and peers. Specifically, within this framework, we explore how beliefs and perceptions related to the sociocultural context affect provider's power within their therapeutic/counseling relationships; how practices and participation reflect norms that influence behaviors and the nature of engagement within the purview of one's roles; the extent to which access to assets influence provider behavior; and how formal and informal rights/norms are affected by health systems structures, policies, and governance.

**FIGURE:**
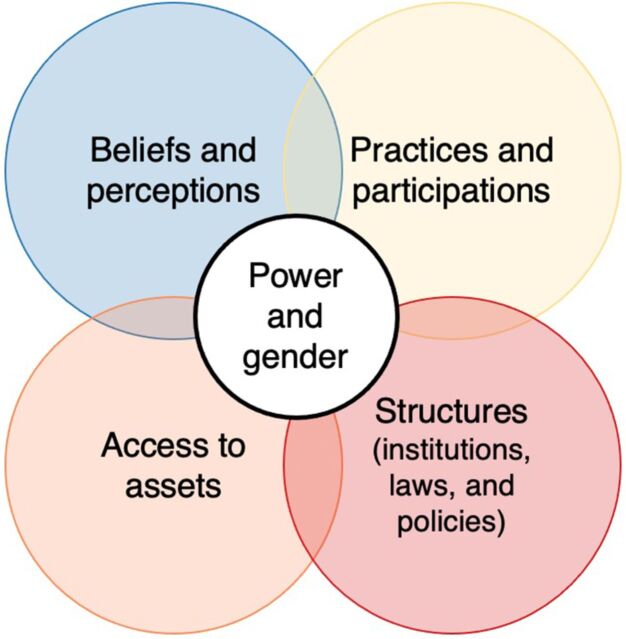
Mapping 4 Domains That Influence Power and Gender Health Care Provider Behaviors and Experience^a^ ^a^Adapted from Betron et al.^19^

To date, there has been limited application of this framework to examine power and gender dynamics among HCPs, and across health areas or country contexts. In our study, we qualitatively explore these interrelated domains across different cadres of HCPs and 4 country contexts (Kenya, Malawi, Madagascar, and Togo) to understand how power is differentially experienced based on one's position and function within the health system. We explore manifestations of power and secondarily gender dynamics in RMNH program settings through 3 generalized cadres, including community-based providers, facility-based providers, and facility-based senior providers/managers.

## METHODS

We conducted a secondary analysis of qualitative in-depth interviews (IDIs, n=123) with HCPs across Kenya, Malawi, Madagascar, and Togo providing RMNH services. The studies focus on varied RMNH services, including newborn/young child hospitalization, critical care for maternal complications (e.g., postpartum hemorrhage [PPH]), and community-level family planning (FP) counseling and service provision. IDIs included information on HCPs' experiences and perspectives on their work, work environment, and interaction with clients and the community.

### Study Settings and Data Sources

This cross-cutting power and gender analysis draws on 4 independent studies in sub-Saharan Africa under Breakthrough RESEARCH, a U.S. Agency for International Development-supported global SBC evidence generation project.

A formative study in Kenya explored the experience of providing care for newborns, infants, and young children aged younger than 2 years to inform the participatory development of a provider behavior change intervention.^21^ Data were collected in December 2019 across 5 hospitals in 2 counties (Nairobi and Bungoma). IDIs were conducted with 32 HCPs, including all cadre types (Table 1), using distinct interview guides (Supplement). Purposive sampling of the range of types of HCPs working in or with inpatient settings with parents of sick young children, including those based in postnatal wards, newborn units, and pediatric wards. Community-based providers included community health extension workers (CHEWs) linked to a facility. Facility-based providers included nurses, midwives, clinical officers, and medical officers. Facility-based senior providers/managers included in-charges, chief nurses, matrons, and specialist doctors. HCPs were recruited by trained research assistants with social science and public health backgrounds after an introduction by the study coordinator.

**TABLE 1.** Health Care Provider Characteristics From Kenya, Madagascar, Malawi, and Togo

	Kenya, No.	Malawi, No.	Madagascar, No.	Togo, No.
Total providers	32	44^a^	35	12^b^
Female providers	23	31	25	5
Male providers	9	12	10	7
Providers by cadre				
Community-based providers	4	0	0	10
Facility-based providers	16	40	29	0
Facility-based senior provider/managers	12	4	6	2
Practice location				
Community	3^c^	n/a	n/a	12
Health centers	n/a	23	26	n/a
Hospitals	29	21	9	n/a

Abbreviation: n/a, not applicable.

a Missing provider sex information for 1 provider.

b All Togolese providers (including senior providers/managers) work in the community.

c These providers work in the community but are linked with hospitals.

Two implementation research studies in Malawi and Madagascar, which were part of the Advancing Postpartum Hemorrhage Care research portfolio, focused on provider behavior change approaches to improve the quality of PPH care.^22^^,^^23^ These studies were conducted in 4 districts in Malawi (Balaka, Zomba, Lilongwe, and Dowa) and 10 districts in Madagascar (Brickaville, Toasmansina I and II, Vatomandry, Ifanadiana, Mankara, Mananjary, Vohipeno, Ikongo, and Mahonoro). Data were collected between December 2019 and February 2021 at 2 time points, with minimal overlap between providers interviewed at baseline and endline. IDIs were conducted with 44 HCPs in Malawi and 35 HCPs in Madagascar (Table 1). Facility-based HCPs managing maternity complications, particularly PPH, in maternity and postnatal wards were sampled purposively at health centers and hospitals. Facility-based providers in Malawi and Madagascar included nurses, midwives, technicians, and clinical coordinators/mentors, and facility-based senior providers/managers included in-charge matrons and specialist doctors. Trained study teams included nursing and public health research staff recruited HCPs.

An implementation research study in Togo centered on engaging men for effective FP through community-based approaches aimed at strengthening couples' communication.^24^ This study was conducted between November 2018 and June 2019 (2 rounds of data collection with minimal participant overlap) in Kpimé and Kpadapé subdistricts, Kloto district. IDIs were conducted with 12 HCPs—all within the community-based provider cadre (e.g., CHWs and community relays) (Table 1). Community-based provider cadres in Togo provide counseling and contraceptive methods, including pills, condoms, and injectables—among other basic health services. HCPs were purposively sampled based on their involvement in the communication approaches and provided either home-based counseling (of a couple or individuals) or group-based discussions. Community-based providers were recruited and interviewed before and after counseling sessions and group discussions by gender-matched study teams.

All participants received information about their study's voluntary nature, associated risks, and benefits and agreed to participate through an informed consent process.

Table 1 presents respondent information across the 4 studies. Half the HCPs interviewed are women, with some community-based and community-linked providers and most HCPs working in facility settings.

### Data Analysis: Framework Application

Secondary qualitative analyses enable the investigation of emergent concepts that were not the focus of the primary studies. They draw out deeper insights for research and programming and increase credibility and transferability of individual studies.^25^^,^^26^ Although these data draw on studies that did not explicitly focus on HCP power and/or gender, these constructs emerged to varying degrees in provider interviews. We apply a framework analysis approach informed by the adapted Betron mapping (Figure), as it is well suited to interpreting cross-sectional descriptive data to generate explanations and recommendations for programs and policy.^27^^,^^28^ The framework analysis approach complements secondary analyses and provides structure to understanding constructs like power and gender.

The data for this analysis included transcripts and varied preliminary analyses by project-specific principal investigators and their country-based research teams in Madagascar, Malawi, Kenya, and Togo. Transcripts had been coded using NVivo.12 or Atlas.ti software. We reviewed code outputs, transcripts, and charts for themes related to power and gender within each health area-specific study (coding any additional material as necessary). Sample codes reviewed include “provider-client interactions,” “drivers of mistreatment,” “quality of care,” “recommendations for improving relationships,” “supportive supervision,” and “policies and guidelines,” among others. Country-specific summary memos were prepared by the second author using an inductive and deductive approach based on a priori themes based on the adapted framework and content emerging from the data. Reliability issues were resolved through a discussion with coauthors, including in-country study investigators.

Applying the Betron et al. mapping alongside inductive data exploration enabled us to iterate on our analytic framework to better situate and synthesize the power dynamics with interspersed gender findings experienced by providers working in varied settings. PS, SP, and SM engaged in a deliberative process of data review to adaptively operationalize the Betron et al. mapping as our analytic framework. We maintained Betron's broad domains and rearranged subthemes resonant with our inductive data exploration. We examined, through memo-writing and generation of a cross-country data summary sheet, several questions per domain (Table 2). Our results structure was informed by consolidating our data summary sheet and memos with a lens toward how HCPs' power to, within, with, and over manifest in varied HCP relationships. We discussed the domains with investigators from each study, including where their study findings placed among the domains, to finalize our framework application and results.

**TABLE 2.** Querying Health Care Providers on Power Across Mapping Domains^a^

Domain	Themes	Data Exploration Questions
Beliefs and perceptions	Perceived provider/community interaction and normalized mistreatment	How do self-reported provider and community/client interactions reveal each perspective's attitude and exemplify relational dynamics between HCPs and clients? In what ways may these relational dynamics be interpretable through normalized mistreatment and/or disrespect?
	Societal beliefs and social norms	Are there societal/community beliefs and practices that act as barriers to/interfere with provision of care? What social norms interfere with provision of care?
Practices and participation	Interprofessional collaboration and mechanisms	Is there respectful interprofessional collaboration between provider cadres? What positive collaboration mechanisms exist?
	Challenging authority	Can midwives or CHWs challenge male or female colleagues and/or senior doctors?
Access to assets	Opportunities for advancement and remuneration	Is there additional remuneration and/or other opportunities for career advancement?
	Access to material/spatial resources	Do providers have access to necessary resources and commodities through supply chains to effectively do their jobs?
	Supervision quality and emotional support	Do varying levels of providers feel supported by the system in which they work? What is the quality of supervision?
Structures (institutions, law, and policies)	Human resource availability and capability	Are there enough human resources and do providers have sufficient skills to carry out their work?
	Policies, protections, and guidance	Are there any policies or regulatory protections in place? How does education, or lack thereof, on the part of the provider and/or community member impact provision of care?

a Adapted from Betron et al.^19^

The manifestation of power dynamics was also explored by gender, when data were available, based on stratified responses (male or female) and as they arose thematically across the 4 domains. We also analyzed the data stratified by practice setting and by HCP type to understand how power variably manifests. Specifically, we assessed patterns—similarities and differences—between community-based providers and facility-based providers responsible for direct services, as well as facility-/program-level senior providers/managers that oversee and support service provision in facility and community settings.

### Ethical Approvals

As a secondary analysis, this study was exempt from Population Council's Institutional Review Board ethical review (#EX2021006); however, the studies from which we drew our data sources were all reviewed and approved by U.S. and country-based ethics review committees. Population Council's Institutional Review Board (p910 and p893), USA, granted approval for studies in Madagascar, Malawi, and Kenya, with local approvals from the Committee on Ethics of Biomedical Research at the Ministry of Health Madagascar, College of Medicine Research and Ethics in Malawi, and the Ethics & Science Review Committee at Amref Health Africa in Kenya. The Togo study was approved by the Georgetown University Institutional Review Board, USA, and the Bioethics Research for Health Committee in Togo.

## RESULTS

Results present providers' perspectives and experiences of power dynamics—including power within, power to, power with, and power over—across 4 domains (Figure). For each domain, we present key themes that highlight dynamics and distinctions by country context, HCP cadre, and/or gender as they emerged.

### Beliefs and Perceptions

The beliefs and perceptions domain captures the beliefs and attitudes that give rise to power dynamics between HCPs and clients/communities. These are underpinned by local assumptions about what it means to be an HCP and a user of health services. Power dynamics, exemplified through interaction, normalized mistreatment, societal beliefs, and discrimination, influenced provider behavior in varied health service contexts across the 4 countries.

#### Perceived Provider-Client/Community Interaction and Normalized Mistreatment

Perceived interactions between HCPs and clients in our sample were often based on individual experiences of power differences and information asymmetries and further influenced by provider/facility reputation in the community. HCP perspectives revealed that although clinical knowledge gave them relative power with clients, the quality of interactions with clients affected their ability to provide services effectively. No gender differences in HCP approaches to interacting with clients emerged in Kenya, Malawi, and Madagascar, and to a limited extent in Togo.

HCP perspectives reveal that clinical knowledge gave them relative power with clients, but the quality of interactions with clients affected their ability to provide services effectively.

Power dynamics manifested in the communication and tone set and perceived between providers and clients during 1-on-1 service interactions. Across all countries, HCP cadre, and gender, we found that strategies, such as listening, coaching/mentoring, and dialoguing that emphasized communication style and tone, could foster a favorable power dynamic between providers and clients. These strategies enabled positive interactions that reduced provider power over clients and elevated provider power to achieve a service goal.

*Listen to them [families of babies], encourage them to ask questions. Let the health worker be enthusiastic to answer, not assume they [families] do not understand. They do. In the process of question, answering, talking, you form a rapport. They gain confidence in what you are doing.* —Female, hospital-based provider, newborn care, Kenya

In Togo, these strategies, alongside trusting HCP-client relationships and HCP age, were more relevant than an HCP's gender in affecting individual perceptions of provider-client interactions. However, there was a slight difference in how female HCPs counseled couples (separately first, then together) compared to how male HCPs approached the interaction (joint session). The following quote supports the influence of age on provider-client interactions.

*In terms of difficulties, some people consider that we're not old enough to speak to them, so it's sometimes difficult.* —Female, community-based provider, FP/reproductive health (RH), Togo

Contrastingly, when HCPs inadequately communicated with or showed disrespect toward, neglected, or verbally abused (“yelled”/“shouted at”) clients, negative power dynamics arose. In maternal newborn complication contexts, for example, these dynamics strained patient-provider relationships, led to reduced urgent care-seeking by the client, and attenuated the provider's ability to prevent adverse maternal newborn outcomes.

*It [the experience] may affect them in future. If the outcome was poor, it may make them fear to seek health care … they will lose trust in me and they will never want to listen to me again.* —Female, hospital-based senior provider/manager, newborn care, Kenya

We found across the studies that community perceptions of provider-client interactions—both 1-on-1 and group service interactions—influenced HCP reputations, which affected prevailing power relations between providers and communities. Our data showed community members' confidence and trust in HCPs working in community and facility settings empowered them to counsel clients. Irrespective of cadre and gender, positive HCP reputations were built and sustained when “good experiences” were shared within communities and providers engaged with clients to foster familiarity over time.

*The midwife is not mean … If you do it [welcome kindly, smile at, and talk] to 1 person, it extends to the whole locality and it is a good advertisement for you: “She's very cool midwife there.”* —Female, health center-based provider, PPH, Madagascar

*Because I work in health, people tend to know me… they are not hesitant when it comes to accepting my appointments with them. So, I don't really have any difficulty.* —Male, community-based provider, FP/RH, Togo

*It is rare to hear negative messages or rumors about counseling activities.* —Male, facility-based senior provider/manager, FP/RH, Togo

Negative reputations or normalized expectations of mistreatment arose when “bad experiences” were shared among clients and prevented future interactions with providers. Although negative HCP reputation appeared less likely of CHWs in Togo, where community-based HCPs shared socioeconomic and living similarities with clients, facility-based providers often described communities' fear that HCPs might inadequately respond to their needs. In Madagascar, Malawi, and Kenya, propagated community fear around potential negative provider-client interactions created a tense dynamic between clients and providers that limited HCPs' ability to provide necessary care. In some cases, providers expressed feeling mistreated by their communities, leading to a transference of HCP stress onto their clients.

*We get accused, “I brought my child on time, this and this happened, you people killed my child,” such things. I wish this community understands that as much as we are HCPs, we are also parents and mothers, and we will not come here to make them suffer … it is hard for [parents] to understand their child was so sick, even if we were to do miracles, your child came in late. They blame us for no reason at all.* —Female, hospital-based senior provider/manager, newborn care, Kenya

We found that some providers were aware of how their behavior was experienced or understood as mistreatment by communities. For example, in Malawi, self-reflection on this point differed by gender. Some male providers adopted a community-blaming approach, and some female providers recognized the provider's role in affecting a client's experience of care.

*We have a community that does not appreciate our services, for instance, there [w]as a time when the radio announced that nurses were beating patients in the labor ward.* —Male, health center-based provider, PPH, Malawi

*We [providers] are really bad with how we talk … The patients are in pain, laboring is difficult. [If] a health worker does not talk nicely to the patients next time, they won't [come] because we've shouted at them.* —Female, hospital-based provider, PPH, Malawi

All HCP cadres in all 4 countries described how a community's societal beliefs and social norms at times misaligned with medical procedures—affecting HCPs' power to provide timely care.

#### Societal Beliefs and Social Norms

All HCP cadres in Madagascar, Malawi, Kenya, and Togo described how a community's societal (cultural and religious) beliefs and social norms at times misaligned with necessary medical procedures—causing friction in an individual provider-client relationship and affecting HCPs' power to provide timely care amid constraints. In Malawi and Togo, the religious belief that contraceptive methods were “sinful” or “against God” constrained facility- and community-based HCPs' power to provide care. In Madagascar, cultural beliefs and practices at odds with clinical guidelines could pose a risk to mothers and newborns.

*I [Midwife] asked to bring the baby's clothes to prepare them because the woman was about to give birth, and I said, “Where are the baby's clothes?” and the answer that came was, “We don't have one … We are really forbidden to prepare for this.” Then I don't know what to do. There was also a time when where I was actually using the state-offered delivery kit, but, when I arrived in the room, she [mother] took off and handed over the loincloth [to cover baby]. It turns out that this is not done because it “wishes harm” on the baby.* —Female, health center-based provider, PPH, Madagascar

In Kenya, traditional practices occasionally superseded allopathic medicine as providers described families' refusals of necessary medical care for sick newborns or seeking alternative care.

*Once, I had a man who believed the child had been bewitched and wanted to go to a native doctor. He [father] later came and told us, the case is not to be treated at the hospital -it is related to their culture at home and his [neglect] of slaughtering a cow after burying his mother. He refused treatment for the baby [against HCP's medical advice]. So sometimes it is the culture or attitude and not failure of communication [between provider and client].* —Female, hospital-based senior provider/manager, newborn care, Kenya

We found social norms that preferred and elevated senior doctors over other facility-based providers (e.g., nurses and coordinators) affected HCPs' power to provide care in the face of constraints. A Kenyan facility-based senior provider/manager (e.g., nurse in-charge) expressed how a family wanted a doctor to perform a procedure that she could perform.

*Sometimes when these babies come in, they may need an intravenous line. This procedure normally is done by a doctor, but as nurses we are trained to come in when the doctor is busy, put an [intravenous], to get medications on time. Somebody may have the attitude . . . . to wait for the doctor … But it is a procedure [nurses] can do.* —Female, hospital-based senior provider/manager, newborn care, Kenya

### Practices and Participation

The practices and participation domain reflects organizational norms of how provider cadres work within their unique roles and responsibilities, collaborate, make decisions, and engage in mechanisms of feedback, including challenging authority—all of which shape HCPs' power with colleagues toward a health service goal.

#### Interprofessional Collaboration and Mechanisms

Power with and over other providers was experienced through interprofessional collaboration in decision-making and care processes as described through norms of working together in a particular context. Providers described the collaboration between cadres on critical cases as being positive but as reinforcing cadre-specific power relations between senior providers/managers and direct service HCPs. We found this in maternal health settings where advanced team-based care may have been required for managing PPH in Malawi. However, cadre-specific power relations might give privilege to senior providers/managers with higher medical education or managerial responsibilities over those closest to client care (i.e., senior provider over CHEW), which could have challenged a facility-based HCP's power to engage.

Providers described collaboration between cadres on critical cases as positive but as reinforcing cadre-specific power relations between senior providers/managers and direct service HCPs.

*First, the midwife assists, if [she] fails, she calls the clinician, and if they fail, they call the doctor, then the gynecologist. The gynecologist makes the final decision that this condition cannot be controlled – so we refer.* —Female, hospital-based provider, PPH, Malawi

*The stress in the execution of my work … often the problem comes from the matron! What she could not do properly I have to do my best to take it back calmly … find out how to achieve this*. —Female, hospital-based provider, PPH, Madagascar

Kenyan, Togolese, and Malawian providers—male and female—suggested that overcoming this hierarchical challenge and improving collaboration between cadres required understanding the unique value and skills each HCP brought to a care team, including their relationships with and proximity to clients/communities. Although community-based HCPs (CHWs and CHEWs) understandably lacked the skills required of nurses and doctors in times of acute emergency, they possessed higher familiarity with their clients, families, and communities. In Kenya, a facility-linked CHEW reiterated the importance of facility-based providers engaging with catchment communities as a way of increasing HCP power to deliver high-quality care for patients.

*All of us, the CHEWs, the health workers work together. I have done the community strategy – going to the household level … When our newer colleagues go to the community … they have to change a little. If [a nurse] trained in Nairobi comes here [rural county] and you take her to [village], there are things that she has to change.* —Male, CHEW, newborn care, Kenya

Male and female facility-based HCPs from Malawi and Kenya concurred that peer support and routine meetings where senior providers/managers and staff focused on “communication channels” and codeveloped “action plans” would bolster collaboration between HCP cadres and alleviate provider stress. Specifically, facility-based HCPs described how these strategies showed promise in distributing power equitably with each other to effectively provide care.

*We receive support from our fellow health workers… there is teamwork; when there is a complication with a woman and you are unsure of what to do, you can consult and the other health workers are always there to help.* —Female, health center-based provider, PPH, Malawi

Sparse data on the mechanisms through which collaboration took place across all 4 countries emphasized spending time and engaging in positive communication between colleagues. A popularly used and well-received feedback mechanism that encouraged interprofessional collaboration included ad hoc and routine meetings between peer providers and supervisors. Additionally, the use of phones to collaborate between HCPs and senior providers was commonly mentioned in Malawi and Madagascar, as often, the senior providers were not in the same physical space.

#### Challenging Authority

Given the HCP cadre-associated organizational norm of deferring to medical authority, we noted that community and facility-based providers had generally low levels of power to challenge senior providers'/managers' decision-making. The ability of lower-level providers to question those who ranked above them was examined in each country. Despite limited data, this low power manifested similarly among male and female HCPs and across cadres in all 4 countries. Few facility-based providers in Kenya and Malawi expressed their power restrained by senior providers as a stress-inducing practice.

*[If] someone says no when you know that it is not the right thing – that stresses you because you know the right way [to manage PPH], but because he or she is a consultant she is saying no it gives you a hard time.* —Female, hospital-based provider, PPH, Malawi

In Togo, while community-based HCPs held less power than facility-based providers, they felt comfortable engaging with authority figures (e.g., supervisors, facility and community nurses, and village chiefs). This was derived from their trusted relationships with clients and the autonomy embedded in their individual (non-team-based) nature of work providing FP/RH counseling in a community setting.

*We are doing okay [in counseling], but if we could get some help to support us, it would be good. A good counselor needs read a lot and have a supervisor guiding him to improve the work … [Also] I involve the village chief by informing him about each session … even just after a training when I wanted to build awareness, the chief himself mobilized people for me.* —Male, community-based provider, FP/RH, Togo

### Access to Assets

Access to assets includes access to material/spatial resources, opportunities for advancement, and supervision quality and emotional support. Who had access to these assets could differentially affect providers' power within and power to provide effective services. We found limited access to assets across HCP cadres and gender in all 4 countries, though non-senior facility-based providers at health centers described it most acutely. Further, our data suggested that even if access to 1 asset enabled providers to enhance their capacity, another might restrict their power to act (e.g., they have some supportive supervision but have no resources).

#### Opportunities for Advancement and Remuneration

Low power within providers manifested in providers' descriptions of limited opportunities for advancement and lack of timely remuneration. We observed this consistently across all HCPs—irrespective of cadre, sex, and work locations (i.e., hospitals vs. health centers). Some facility-based providers in Kenya described stagnating in their jobs for years without growth opportunities as demotivating. Opportunities beyond promotion, such as mentorships and training programs, could motivate and nurture facility-based HCPs, particularly nurses. Yet, these were held ad hoc and could be financially prohibitive to attend, as seen in Madagascar, Malawi, and Kenya.

Some facility-based providers in Kenya described stagnating in their jobs for years without growth opportunities as demotivating.

Facility-based providers and senior providers/managers from Madagascar, Malawi, and Kenya noted delays in payroll disbursements, and the voluntary community-embedded cadre in Togo had different expectations of remuneration (e.g., snacks, water). Compensation/remuneration was mentioned as a motivational asset by all facility-based HCPs of all cadres and sex—with little variability in tone.

*There is no motivation in relation to the salary received. The salary amount of is far from the volume of work we do … [although] you are very serious, sometimes you are a little sleepy because of the lack of motivation and remuneration.* —Male, health center-based provider, PPH, Madagascar

Beyond inadequate remuneration, voluntary community-based HCPs in Togo expressed feeling underappreciated by the health systems in which they worked. This led to perceived unfair treatment with respect to CHWs' work compared to that of facility-based providers, placing higher value on the latter.

*What I don't like as much is the fact that the work of a CHW is really heavy. We are the ones doing all the upfront work before people come to see a [facility-based] provider, but we are not treated as we should be. That is discouraging.* —Female, community-based provider, FP/RH, Togo

Female providers, particularly nurses, struggled with and required additional support for balancing their intersectional gender roles in their professional and personal lives. A female nurse in Kenya reflected on her limited and delayed compensation in relation to her work and her role as the primary income earner and caregiver at home.

*It is about the employer … because a happy employee does things … I am [a] human being, a nurse, with a family to feed. [If] you do not pay me in time or well, as much as I am giving care, I am not comfortable. How can I give a good quality care … knowing that when I go home, I am unable to afford supper for my kid … I see myself as a failure to my children*. —Female, hospital-based provider, newborn care, Kenya

HCPs across all 4 countries described feeling that an extreme lack of material and spatial resources limited their power to effectively care for clients.

#### Access to Material/Spatial Resources

HCPs across all 4 countries (least in Togo) described feeling that an extreme lack of material and spatial resources limited their power to effectively care for clients. We found that limited medicines and lack of basic and complex medical equipment (e.g., gloves, soap, stethoscopes, thermometers, intrauterine devices, and resuscitators) constrained facility-based workers in Kenya (at hospitals), Madagascar, and Malawi (at hospitals and health centers). In contrast, Togolese community-based HCPs described sufficient counseling tools (e.g., job aids) but limited logistical and material support when meeting clients living in remote areas. In Madagascar, health center-based providers mentioned additional challenges with the lack of basic utilities (e.g., water and electricity) compared to hospital-based providers. In Malawi and Madagascar, some HCPs—all cadres and both genders—suffered greater resource strain at the health center level, as described by male and female nurses.

*The equipment used daily at the health center are all obsolete … The materials are worn, beds all broken and even the most minimal objects—step ladders [are broken].* —Male, health center-based provider, PPH, Madagascar

*… sometimes we don't have the materials, we improvise using other things to work effectively.* —Female, health center-based provider, PPH, Malawi

*During the rainy season, we suffer a lot to do the visits. After several complaints, we were given overcoats. There is also distance; we have to travel kilometers to a household and sometimes, if the couple in the household is not there, it totally discourages you.* —Male, community-based provider, FP/RH, Togo

We found that provider stress, influenced by the resource environment, affected their power to function optimally without transferring that onto their clients. HCPs in Madagascar described a lack of staff, amenities, space, and salary combined with high patient volume—a combination that resulted in little time for HCPs to build rapport with patients and ensure comfort. Instances where transference of provider stress to clients occurred left patients and communities feeling dismissed and unheard—in Kenya and Malawi, the pervasiveness of these sentiments normalized poor quality. HCPs reflected that they and their clients were so accustomed to inadequate resources and high client volume that the resultant stress-induced poor quality of care was normalized in the community.

*Some of them [families] do not even understand that it is poor quality. They think it is just normal.* —Female, hospital-based provider, newborn care, Kenya

#### Supervision Quality and Emotional Support

Across countries, cadres, and gender, supervision quality and emotional support emerged as salient themes that affected providers' agency (power within), power to work with peer HCPs, and ability to interact with clients effectively.

HCPs noted supervisor availability and responsiveness, including unlocking resources and providing technical feedback, were key components of supervision quality across all health areas and countries. Some facility-based providers in Malawi, irrespective of practice location, felt supported by their supervisors in resource requests—if they needed something, they were able to ask for it. In Kenya, facility-based providers felt that senior providers/managers should be doing more to provide supportive supervision and ensure timely and adequate dispersal of resources.

*Being a team leader comes with the rules and responsibility of making sure that the rest of the team are fully equipped in terms of knowledge, skills, and the necessary things they need to be able to offer services.* —Female, hospital-based senior provider/manager, newborn care, Kenya

In contrast, in Togo, community-based providers reported positive and active feedback, including moral and technical support, from supervisors who offered encouragement during trainings, back-stopping during community education sessions (e.g., answering questions beyond the HCPs' capacity), as well as provide feedback through routine mechanisms (e.g., individual meetings and reports).

In Togo, community-based providers reported supervisors giving positive and active feedback, including moral and technical support.

*When I ask him [supervisor] to assist me, he does it without hesitating, so I can say that my supervisor supports me in my activities. There is a nice relationship between us.* —Female, community-based provider, FP/RH, Togo

Emotional support, although not always available, was expressed as enhancing provider power within facility-based critical care settings. We found that Malawian providers experienced burnout and frustration while trying to work. These providers described feeling supported emotionally by peers and supervisors rather than facility management. Providers in Kenya commiserated about feeling overwhelmed and expressed a need for better mental health support for those working in high-stress health areas. In both countries, emotional support interventions offered mechanisms to empower providers in their balancing work and stress (e.g., nurturing power within).

*When something affects your psychological well-being, it interferes with your work and your home relationships, so there is need to have mental health personnel to assist … We have seen providers just shout at anyone at home without reason because of the stressful situations they had at work…having psychological debriefings is vital.* —Female, health center-based provider, PPH, Malawi

*We have a big gap in management of emotional stress, we don't have a psychologist so that's an area of big gap even for us health providers.* —Female, hospital-based senior provider/manager, newborn care, Kenya

Across the 4 countries, there was little explicit mention of how being a female provider or supervisor influenced supervision quality or emotional support. We found in Madagascar and Kenya that HCPs recognized the need for extra sensitivity and support for cross-cadre female providers who were balancing their intersectional caregiving roles at work and home.

*The provider has her own small baby to take care of day and night, we come to annoy her in the middle of the night, so we must help her as best as possible.* —Female, health center-based provider, PPH, Madagascar

*The health care provider needs some time off. If we have correct nurse to patient ratio … [then] the nurse has time for the mother. That is not possible here. You [nurse] go to work, do the cleaning, give drugs, make beds … [and] it's us doing the technical work.* —Female, hospital-based provider, newborn care, Kenya

### Structures—Institutions, Laws, and Policies

Structures, underlying institutional and workforce policies, and rules, though not always transparently communicated, indirectly affected power relations between HCPs and quality care.

*My role is to ensure that patients get quality care by close supervision of the nurses to the students here … to ensure they are carrying out according to the set standards and procedures [of the ward].* —Female, hospital-based provider, newborn care, Kenya

We found that power manifested in structures through the scarcity of human resources and limited policies, protections, and practice guidance, with little difference by HCP cadre or gender.

#### Human Resource Availability and Capability

Facility-based providers in Madagascar, Malawi, and Kenya described human resource shortage among all cadres as a structural challenge to teamwork and sharing of power. In these settings, few providers covered large patient volumes, which could lead, even in resource-rich moments, to suboptimal care quality.

Facility-based providers in Madagascar, Malawi, and Kenya described human resource shortage among all cadres as a structural challenge to teamwork and sharing of power.

*If 1 care provider is going to run a clinic with 30 to 40 patients … we don't have enough time to [provide] dignified care and [cater to the] needs of these children … because of time shortage because you end up doing, clearing and forwarding … it's a challenge - you don't have 1-on-1 attention.* —Female, hospital-based senior provider/manager, newborn care, Kenya

The scarcity of sufficient training limited power within/provider's ability. For example, male and female providers in Malawi and Kenya described lacking technical knowledge and communications skills. There were limited data in Madagascar and Togo to investigate this. Sometimes, in cases of staff turnover, the data showed that new hires created more burden than they alleviated for senior providers/managers because of oversight responsibilities and training requirements. In Kenya, HCPs lamented that mistakes could create more work for supervising and collaborating providers. Malawian supervisors described an inability to exert power within their roles—balancing their coordination and mentorship role with time required for formalized in-service clinical and reporting skill-building. In Madagascar, a facility-based HCP described limited and additional human resources as perpetual problems without recourse.

*He [supervisor] gives advice on the organization of work but in our locality in the countryside, this is not really effective … We cannot do otherwise because it is a problem of insufficient staff … human resources … and it would always persist no matter what you do.* —Male, health center-based provider, PPH, Madagascar

#### Policies, Protections, and Practice Guidance

Providers' power over their interactions and practices in the facility was derived from the institutional policies, protections, and guidance in place. While health area-specific technical and operational policies and protocols existed to some degree across country contexts, there was little mention of provider protections or institutional mechanisms to guide practice. We found Kenya's role distinction guidelines within a newborn unit were clear, while Malawi's conflict resolution, referral, and outdated PPH protocols challenged HCPs' ability to care for women with PPH. A subset of HCPs described the downstream effects of staff rotation policies in Kenya. Provider transfers to units in which they had little to no experience endangered patients and increased HCP vulnerability and stress.

*It doesn't matter if it is a consultant, doctor, or a nurse, there should be a hospital protocol. For example, when a patient comes, it doesn't need a consultant or doctor to tell you to do this … there must be a guideline to follow.* —Female, hospital-based provider, PPH, Malawi

*I have experienced someone working at the ward … or someone who isn't supposed to deal with a newborn is brought to the newborn unit … now she wants to catch up because this is not an area that she's trained in.* —Male, CHEW, newborn care, Kenya

A few Kenyan HCPs reported lacking quality-of-care policies to guide them on how to communicate with parents about their child's care. Others noted that although guidelines existed at the facility level, limitations in the access to assets domain constrained HCPs' power to integrate quality-strengthening programs in practice.

*The guideline is that we discharge babies at 2 kilos … some facilities discharge earlier because of crowding within the facilities. Spatial limitations makes implementation of baby-friendly initiatives a bit difficult, but we try.* —Female, hospital-based senior provider/manager, newborn care, Kenya

Translation of policies into practice affected provider power and capability to practice. We found that HCPs across the 4 countries felt continued professional development would ensure quality care provision—irrespective of cadre and gender. In Kenya and to a lesser extent Malawi, where attending continued professional development sessions was an accepted facility norm, providers noted the importance of free trainings for updating HCPs on the latest guidelines. They also described that translation and dissemination should expand beyond hospitals to health centers and could be a slow process.

*We need to be updated now and again in knowledge and new skills because there are new protocols. This facility that we are [in, there are] a lot of providers … it takes time for someone to improve [their] skills.* —Male, hospital-based provider, PPH, Malawi

## DISCUSSION

Our findings confirm that, for HCPs, power dynamics that underlie RMNH service interactions were manifest in and affected by 4 overlapping domains: beliefs and perceptions, practices and participation, access to assets, and structures. An HCP's power to achieve high-quality care—including counseling and direct service provision—drew on all 4 domains given the range of relationships and interactions involved, including with clients, families, peers, and supervisors. HCPs' power to provide quality care drew primarily from the beliefs and perceptions domain—the more respectful, equal, and open the individual and collective interactions perceived by clients and communities, the more favorable the power dynamic to optimize care experiences. Limited access to assets—including internal and external motivators, material resources, and supervision quality and emotional support—affected HCPs' power within and to effectively deliver high-quality care. Power with and between colleagues to provide services primarily reflected the practices and participation domain—HCP relationships with other providers that gave rise to decision-making and collaboration norms in care provision—as well as the structures that formally and indirectly influenced HCPs' working contexts. Our findings point to the importance of enhancing HCP power with communities, peers, and senior managers as programmatically relevant for improving the quality of care across health areas.

A HCP's power to achieve high-quality care—including counseling and direct service provision—drew on all 4 domains given the range of relationships and interactions involved.

Our study shows varied patterns of how power manifests among community- and facility-based providers and transfers across health areas. These differences ought to be interpreted within cadre-specific scopes of work. In our study, community-based HCPs focused on routine counseling, and facility-based HCPs worked in acute care. Although all HCPs cadres have limited power as measured by access to assets and structures and seen elsewhere in respectful maternity care and community health systems literature,^29^^–^^31^ their power dynamic varies within the beliefs and perceptions and practices and participation domains. Consistent with other studies, community-based HCPs in Togo demonstrated high power to provide services, deriving from their agency to work autonomously in community settings with program support and trusting relationships with clients.^24^^,^^32^^,^^33^ In contrast, facility-based HCPs in Kenya, Madagascar, and Malawi providing services within teams in hierarchical professional environments experienced constraints to their power to practice. This reflects normalized negative facility reputations and deference to authority (senior providers/managers) and restriction of interprofessional collaboration, expanding on similar patterns in the respectful maternity care literature.^34^ Facility-based senior providers/managers hold high power in decision-making authority deriving from their role/position within an institution/program despite their limited direct interaction with clients. HCPs working in health centers have less power than those in hospitals, given exacerbated challenges of limited material resources and opportunities for growth.

Beyond the 4 domains, persisting power dynamics identified in our study concur with other studies and have implications on quality (experience and provision) of care. The diversity of HCPs exercising their power—within, to act, with, and over—triangulates with categories of discretionary and authoritative power identified by Lehmann and Gilson.^14^ Similar to this study, our study suggests that HCPs experience and act upon these types of power variably—including cases where those with less authoritative power (within a hierarchical health system) may have higher discretionary power to provide high-quality care and point to a positive client experience. Our interprovider dynamics-related findings further suggest that too much HCP power over can be as consequential as a lack of power (within, with, and to) to providing quality care. Though our secondary analyses drew on HCP perspectives only, congruent themes and results from other studies corroborating data sources, including client perspectives, suggest the importance and association of equitable power relations with higher quality accountable care.^15^^,^^31^^,^^35^^,^^36^ Future primary studies investigating power and quality of care may consider applying a variety of critical approaches and tools, including but not limited to the power cube (which uses 3 dimensions that bound and shape citizen power and action), root cause analyses (includes several techniques to elicit quality and performance gaps), and case studies (which elaborate on perspectives of power in health policy or program across several actors).^20^^,^^37^^,^^38^

Our findings suggest that power offers a helpful lens to recognize consistencies in how HCPs perceive, internalize, and react to a range of relational and environmental stressors. Provider stress cuts across all 4 domains (e.g., manifests in negative interactions with clients, derives from uncollaborative or resource-deficient environments, exacerbated by rotation policies or norms that place them in wards where they have little experience), affecting providers' power within, namely their self-efficacy and motivation to act. Beyond the need for resources and technical skill development, these findings contribute to a growing urgency to support the mental health and emotional needs of HCPs.^39^^,^^40^ Stress mitigation strategies have been found to vary across HCP cadres. Community-based providers require access to material resources, transport, growth opportunities, and moral encouragement,^41^ but facility-based providers working in critical care settings additionally require more formal psychosocial support options. Future intervention programs for facility-based providers should consider integrating psychological debriefing, counseling, and stress management, among other coping strategies.^42^

Our findings suggest that power offers a helpful lens to recognize consistencies in how HCPs perceive, internalize, and react to a range of relational and environmental stressors.

Gendered perspectives and intersections marginally emerge in the access to assets domain, where inequitable support may uniquely affect power and behaviors of female providers. Our study begins to show that nurses have intersectional roles as women and HCPs, subject to gender biases in the community and facility, and lack sufficient support for childcare. Female nurses may be particularly vulnerable to workplace-induced mental fatigue, requiring additional support.^43^ Although we lacked data to discern comparative gender dynamics as they intersect with HCP power, the findings start to converge with the current body of literature—that shows female CHWs and facility workers experience low pay, few growth opportunities, and limited child support, protection, and power at work and at home.^44^^,^^45^ Although power domains did not vary across female and male interviewees in this secondary analysis, further research is recommended to fully investigate gendered perspectives.

Our study analyzing qualitative data from HCPs in 4 countries points to several implications for provider behavior change program integration and evaluation of power-enhancing approaches. We recommend district health administrators consider strengthening participatory engagement and feedback mechanisms between communities and facilities through social accountability approaches linked to facility-based quality improvement teams.^5^^,^^46^^,^^47^ Together, these promote participation of communities, encourage provider listening, and nudge responses from health management teams. Our data suggest local administrators, HCPs, and clients codevelop and implement norms-targeted structural changes and codes of conduct for quality care that should be further tested through implementation research.^44^^,^^48^^,^^49^ Given the high proportion of nurses/midwives in our HCP sample and in line with other research, nurse-led team-building strategies, such as weekly or monthly meetings, may reinforce mutual respect among HCP cadres, improve interprofessional collaboration, and elevate low-powered facility-based HCPs voices.^50^ These processes allow for judgment-free problem-solving, may motivate challenging authority without fear of retribution, and enable safe spaces that promote positive social and facility norms that mitigate providers' behavioral constraints.^51^^–^^53^ Facility and community health program managers can consider dynamic information and guideline diffusion strategies (remote and in-person) and institutionalization of continued professional development to onboard and sustain HCPs over time. Leveraging civil society partnerships within diffusion strategies and using culturally acceptable, health area-specific, interpersonal communication-promoting job aids within provider training and client interactions may facilitate opportunities for HCPs to realize their power in practice.^50^^,^^54^

### Limitations

This study has several considerations that affect and limit its interpretation. Given our secondary analysis approach, the data were not collected with the aim of investigating power and gender and emerged out of multiple study designs and purposive country selections based on Breakthrough RESEARCH's existing provider behavior change portfolio. Moreover, the adapted Betron framework is applied post hoc in this secondary analysis, and several subthemes within the framework merit further investigation (e.g., challenging authority and interprofessional collaboration). The richness of the data on power and gender themes varied by country project, given the differences in study aims, instruments, and open-endedness of questions. In contrast to Malawi, Madagascar, and Togo designs, the Kenya study used a critical orientation that enables drawing inferences about power (e.g., institutional ethnography); this may have affected the primary analysis preceding our secondary interpretation. This led to a lot of detailed power-related information from Kenya embedded within aspects of care experience at the provider level. Additionally, though power manifested and influenced all provider types, community-based HCPs in the Togo study had less advanced clinical skills than the facility-based cadres in the other 3 countries. Further investigation of power dynamics at the community level is recommended, particularly given the high discretionary power (i.e., power to) of this group and its vulnerability if the community-based providers' scope were to expand. All studies had less information on gender than power; future primary research should consider investigating how power among subgroups of the community and facility providers intersects with and varies by gender. Our study was unable to assess the psychological underpinnings of why power differentials exist among and between HCPs clients but recognizes this as integral to unpacking and programmatically mitigating their negative consequences, particularly within the beliefs and perceptions domain.

Several other study limitations exist. While endline data collection in Malawi and Madagascar occurred during the COVID-19 pandemic, the particular stresses and power dynamics of working in a pandemic situation were not reflected in our data and warrant further attention. We collapsed the pre-post study data collected in Malawi, Madagascar, and Togo, given the interventions were not focused on directly addressing power or gender at the provider level, nor were the data sufficient to tease out any indirect associations. While our study's scope precludes prioritizing types of HCP power for intervention, we recommend future studies consider assessing this as part of the process of informing program approaches using implementation research. Additionally, a limitation of secondary analyses is the layer of separation between the researchers of this analysis and participants in the studies this analysis draws on. To overcome this challenge, we collaborated with study investigators and drew on the perspectives of data managers to ensure adequate contextualization of our findings and recommendations.

## CONCLUSION

Our findings suggest that prevailing power and gender dynamics in varied relationships at the health service interface influence providers' behaviors and clients' care experiences. They also suggest that applying power, and secondarily, gender lenses can elucidate consistencies in how providers perceive, internalize, and react to a range of relational and environmental stressors. Although enablers of power and positive client experience center around trusted and respectful engagement among HCPs and between HCPs and clients, constraints derive from restrictive or shifting institutional policy and limited access to resources, advancement opportunities, and supportive supervision. Moreover, too much HCP power can be as consequential as a lack of power to providing quality care.

Unveiling these dynamics can elevate HCPs' perspectives on power-enhancing approaches to strengthen quality of care and generate more nuanced SBC programming interventions aimed at better supporting HCPs. Program and facility stakeholders should consider participatory mechanisms between communities and HCPs as well as targeted team-building and emotional support SBC interventions to enable equitable power dynamics and improved provider and client experience of care.

Finally, ample scope for research and learnings around power remains. Specifically, we recommend further investigation of the psychological underpinnings of power, the use of theoretical frameworks, critical approaches, and tools to assess power and quality of care. In addition, we recommend an ongoing focus on implementation research to evaluate power differences and dynamics in response to SBC programming.

## Supplementary Material

GHSP-D-22-00420-supplement.pdf

## Notes

**Peer Reviewed**

**First published online:** August 24, 2023.
