# Supplementary material for: Applying a Power and Gender Lens to Understanding Health Care Provider Experience and Behavior: A Multicountry Qualitative Study
Source: Glob Health Sci Pract. 2023 Nov 30;11(Suppl 1):e2200420. doi: 10.9745/GHSP-D-22-00420 (PMC10698231; doi:10.9745/GHSP-D-22-00420)
Supplement: GHSP-D-22-00420-supplement.pdf [file GHSP-D-22-00420-supplement.pdf]

Supplement to: Sripad P, Peterson S, Idrissou D, et al. Applying a power and gender lens to understanding health care provider experience and behavior: a multicountry qualitative study. *Glob Health Sci Pract*. 2023;11(Suppl 3):e2-00420. https://doi.org/10.9745/GHSP-D-22-00420

SUPPLEMENT. INTERVIEW GUIDES FROM STUDIES IN KENYA, MADAGASCAR, MALAWI, AND TOGO

Kenya

In-Depth Interview Guide For Policy Makers

1. Which policies exist to support respectful care during childbirth, newborn care, young children and postnatal care services in the country? In this county?
2. Who is the caregiver? Who makes these decisions? Where do most parents/caregivers take their newborn or young infant if there is a problem?
3. What affects parents/care-giver decisions to seek childbirth, newborn, young children and postnatal care services at facilities in this county?

We have heard from prior studies that respectful care plays a role in care-seeking decisions of parents/care-givers and would like to discuss the relationship of parents/care-givers and service providers when they visit a health facility for newborn care, postnatal care, or for a sick child needing hospitalization.

4. How do parents/caregivers typically interact with providers at health facilities in this county?
5. What does mistreatment/disrespectful newborn and young infant care look like in this county? How differently does it manifest at various level of health care?
6. What does respectful newborn and young infant care look like in this county? How differently does it manifest at various level of health care?
7. What promotes or limits integrated family centered respectful newborn care and young infant (provider training, family/caregiver's awareness, health system structures and process, policy, protocols and guidelines)?

We want to explore what the concept of family centered care looks like in this context.

8. What do you know about family centered care? What do you think this means for newborn and young infant care in this county?
9. How does early newborn care, routine PNC, and young infant care function? How can these platforms be strengthened to include integrated respectful newborn care?
10. How best would families/care givers, providers and health managers promote/implement family centered newborn care and young infant? What can be done at the policy level? At the county level? At the facility level? At the community level?
11. How would we adequately monitor the proposed intervention/interventions to promote family centered care and reduce incidences of newborn and young infant disrespect at health care facilities?
12. Probe for examples of family centered approaches such as KMC, *we want to explore to what extent Kangaroo Mother Care approach is used in this county What* do you know about KMC breast feeding, rooming in?
13. What is your experience regarding caring for young infants and sick newborns in this county?

Supplement to: Sripad P, Peterson S, Idrissou D, et al. Applying a power and gender lens to understanding health care provider experience and behavior: a multicountry qualitative study. *Glob Health Sci Pract*. 2023;11(Suppl 3):e2-00420. https://doi.org/10.9745/GHSP-D-22-00420

KENYA
In-Depth Interview Guide For Health Professionals

1. Which policies exist to support respectful care during childbirth, newborn care, and postnatal care services in the country? In this county?
2. What about care of sick children up to 2 years of age?
3. Who is the caregiver? Who makes these decisions? Where do most parents/caregivers take their newborn, infant or child if there is a problem?
4. What affects parents/care-giver decisions to seek childbirth, newborn, young infant and postnatal care services at facilities in this county?

We have heard from prior studies that respectful care plays a role in care-seeking decisions of parents/care-givers and would like to discuss the relationship of parents/care-givers and service providers when they visit a health facility for newborn care, postnatal care, or for a sick child needing hospitalization.

5. How do parents/caregivers typically interact with providers at health facilities in this hospital?
6. What does mistreatment/disrespectful newborn care, infant or young child services look like in this hospital? How differently does it manifest at various level of health care?
7. What does respectful newborn and young infants care look like in this hospital? How differently does it manifest at various levels of health care?
8. What promotes or limits family centered care for newborn and young infants (provider training, family/caregiver's awareness, health system structures and process, policy, protocols and guidelines)?

We want to explore what the concept of involving families in the care for young children looks like in this context.

9. What do you know about involving parents in their child's care? What do you think this means for all newborns? for young sick children up to age 2?
   - What do you think it means for small and sick newborns and sick infant care in this county? At this hospital?
   - What about the older child up to 2 years?
10. How does early newborn care, routine PNC, and young childcare function? How can these platforms be strengthened to include respectful childcare?
11. What do you know about KMC, cognitive development breast feeding, nutrition/feeding for young infants and newborn?
12. What is your experience regarding caring for small and sick newborns and young children in this county?
13. How best would families/care givers, providers and health managers promote/implement family centered care for newborn and young infants care? What can be done at the policy level? At the county level? At the facility level? At the community level?

Supplement to: Sripad P, Peterson S, Idrissou D, et al. Applying a power and gender lens to understanding health care provider experience and behavior: a multicountry qualitative study. *Glob Health Sci Pract*. 2023;11(Suppl 3):e2-00420. https://doi.org/10.9745/GHSP-D-22-00420

MALAWI AND MADAGASCAR

In-depth interview guide for providers in maternity units

Questions	Probe (s)
**Q1**. Please describe the general issues around maternity facilities that you have seen over time while providing service to pregnant women?	What are your views on maternity facilities (including maternity waiting homes in Malawi) regarding women seeking to stay nearby before delivery? **[Probe for how they have been run in the past and if any changes and why, nature of changes]**
**Q2**. What are the contextual issues that may be facilitating or constraining use of maternity services?	Probe for contextual factors constraining service coverage, utilization or quality- **Are supplies and commodities, human resources, management or governance factors others? If so which ones?**<br><br>**Enabling environment**-, supplies, supports and tools-, health teams and management, enabling environment and support services<br><br>**Financial factors** for health staff or clients<br><br>Recent changes in social, cultural, economic or physical context<br><br>Of all factors which is the most significant changes or outcomes and reasons for ranking<br><br>**What can be done to improve/ensure the facilities respond well to the management of maternity health care services**
**Topic II: Cost of accessing facility care**	
**Q5.** What are the current cost of accessing facilities?	[**Probe for what costs do women and their families incur if a women stays in a maternity facility? - what do they pay for ?-- commodities, fees, other payments (under-the-table fees)? – if payment were made for what and how were payments made]**<br><br>Other social costs? Any changes that has been observed over time?<br><br>[**Probe for whether women perceive other costs - time away from families, who takes care of other family members, separation, economic activities at home**
**Topic III: Recommendations**	
**Q7** What suggestions do you have for improving the maternity facilities at the facility level?	What are the ways in which pregnant women and postpartum women's can be improved?<br><br>What areas of improvement are needed? **[linkages with facility, management, others]**

Supplement to: Sripad P, Peterson S, Idrissou D, et al. Applying a power and gender lens to understanding health care provider experience and behavior: a multicountry qualitative study. *Glob Health Sci Pract*. 2023;11(Suppl 3):e2-00420. https://doi.org/10.9745/GHSP-D-22-00420

MALAWI AND MADAGASCAR

Maternity Unit Provider In-depth Interview Guide

Overview of factors affecting utilization of health services during childbirth and general providers perceptions on childbirth health services

1. What do you feel is the biggest problem in maternal health in this area? (Probe: complications, ANC, PNC, nutrition, delivery care, health systems limitations?)
2. What are the greatest problems or challenges providers face when providing services to women during childbirth in this community? Why do you say so?
3. What factors do you believe affect the decision of where pregnant women seek delivery services during childbirth (either at facility or use of TBAs)? What makes you think this way?

Exploring possible stressors for maternity unit providers

4. From your perspective, what factors affect your ability to do your job

Probes*: Give examples of what helps you to provide services. Give examples of what can hinder your ability to provide services. How does your work environment affect your performance? Give positive /negative examples*

5. Can you think of a time that you felt stressed about your work in maternity? Please describe

Probe*: What do you think led to this feeling? Anything else? Is there anything that might have helped to alleviate the stress you felt? How might your supervisor have helped you in that situation?*

Now I am going to ask about your understanding of factors related to PPH prevention, detection, and management

6. In your opinion, what is the relative burden of PPH in this country and what is the impact that these conditions have on maternal and child health?
7. Are you aware of any government policy or commitment to address the situation? If yes, could you please describe it?
8. What do you know about PPH? How do you determine if a woman has PPH? What are you views on how this could be prevented and treated? Please describe the main responsibilities in your role and the challenges you face in terms of treating/supporting women with PPH?
9. Are there any guidelines you refer to when doing your work? How useful are they? What do you usually advise women about PPH?
10. How do you manage women with PPH? Please can you describe what happened the last time you managed a woman with PPH? *Probe: What does the referral process look like? Why do you refer women with PPH Please describe.*
11. What would you suggest is needed in order to bring the necessary care to these women?
12. Are you aware of any strategies to help women in the community who have PPH and cannot reach a facility? (Probe: misoprostol, referral)

Supplement to: Sripad P, Peterson S, Idrissou D, et al. Applying a power and gender lens to understanding health care provider experience and behavior: a multicountry qualitative study. *Glob Health Sci Pract*. 2023;11(Suppl 3):e2-00420. https://doi.org/10.9745/GHSP-D-22-00420

TOGO
In-depth interview guide for CHWs and Facility-based Health Providers

SECTION 1. Background and role as provider

I would like to start by learning about what you do here as a CHW or service provider

1. Could you tell me a little bit about yourself and your role as a [CHW] or [provider]? What kinds of things do you do?
2. What do you like about your job? What are things that you do not like as much?

SECTION 2. *Pre-Counseling*: Experiences with training

3. How did you start implementing and/or supporting the couples counseling activities? Probe:
   - → Was it clear to you what was expected of you after the training?
   - → What was easy about starting the activities? What was difficult?
   - → What are people saying about couple counseling/male engagement?
   - → Do you adjust any of the activities? If so, why and how did you adjust some of them?

[FOR CHWs] SECTION 3a. *During Counseling:* Experiences with the couples counseling sessions in the community/ *Now let's move on to some questions about your work with couples.*

4. I would like you to think about your experience and interaction with participants and/or couples within the counseling sessions. What was it like working with the couple/s during sessions and/or visits? Probe:
   - → What kinds of things worked well when working with the couples?
   - → What kinds of things did not work well? Please explain.
   - → What about the materials? How did you use them?
   - → Which materials did you find more or less useful? Why was that the case?
   - → How did you feel delivering counseling to women alone, versus women with their husbands during the sessions? In what ways did women participate? What about men?
   - → How did you feel sharing the information on the different topics *(list the 4 topics)*? What was easy? What was difficult?
   - → What challenges did you have during group counseling? Were you able to resolve them?

5. What was your experience being a facilitator/ couples counselor? Probe:
   - → What was most challenging? What was easiest?
   - → How long would it normally take you to prepare for each of the session activities (e.g. hours and/or times per week)?
   - → How long would it normally take you to deliver the activities? Was it different for when women were alone versus couples together?

6. What kinds of skills do you think are needed to be a good counselor or facilitator for sessions with couples? Probe: For example, about communication, facilitating, listening, etc.

7. How supported did you feel when conducting these activities? Probe:
   - → From your health facility supervisor? And/or iNGO staff? Please explain.
   - → How often did you communicate with your facility supervisor about the counseling activities?
   - → What kinds of things did you talk with your supervisor about related to the couples counseling activities?
   - → Did you feel you had the appropriate guidance, tools and resources to implement the activities? Please explain.
8. Were there any couples participating in the sessions who expressed interest in using family planning methods (e.g. pills, injectables, implants, etc)? Probe:
   - → Did you refer these couples to a health center? How did that work?

Supplement to: Sripad P, Peterson S, Idrissou D, et al. Applying a power and gender lens to understanding health care provider experience and behavior: a multicountry qualitative study. *Glob Health Sci Pract*. 2023;11(Suppl 3):e2-00420. https://doi.org/10.9745/GHSP-D-22-00420

- → Can you give me an example or a story of a couple who went to the health center after the counseling session? What happened afterward?
- → How did you follow-up, if at all, with couples, women and/or men who went to the health center to seek more information about FP?

9. What are some changes you have seen in your community, if at all, as a result of the couples counseling sessions? Probe:
   - → For example, around couple communication? And/or conflict among couples?
   - → Use of family planning services? And/or beliefs around family planning methods
   - → Use of services in general by men? Among couples?
   - → Have there been any negative consequences?

[FOR PROVIDERS] SECTION 3b. *After counseling.* Receiving clients from couples counseling activities. *Now let's move on to some questions about your experiences with the couples counseling activities*

10. What was your experience supervising the CHWs delivering the new couples counseling activities? Probe:
    - → What was easy about the work? What was most challenging?
    - → How often did you communicate with the CHW? What kinds of things did you talk about?
    - → In what areas did they need most support?
    - → Did you feel you had the appropriate guidance, tools and resources to supervise and support the activities? Please explain.
11. What skills do you think are needed to be a supervisor of the couples counseling activities? Probe:
    - → For example, about mentoring, or communication, et al.
    - → Are these different than the skills needed to supervise other forms of family planning counseling?
12. Where there challenges with couples/women obtaining the methods they desired after counseling? Could you say more. Probe:
    - → Does the health center ever have stock outs? How often? Which methods? And when?
    - → Are there certain family planning methods that clients usually prefer? For what reasons?
    - → How many clients do you usually counsel per month? How many take home a method?
13. Has anyone talked to you about obtaining a family planning method after being referred by a CHW during couples counseling sessions? Probe:
    - → Was it just women? Or couples? Did the clients leave with a method?
    - → Can you share a story or example of a woman, man and/or couple?
    - → For which methods? How did you know they were sent by the CHW?

SECTION 4. CHW and Provider behavior change

Thank you for your answers so far. These questions are about what it means to you to be a competent counselor and/or facilitator.

14. I'd like you to remember when you first became a [CHW] or [provider]. What motivated you to do this job? What motivates you now? How has changed over time, if at all? Does being in new strategies to engage men influence your motivation at all?
15. In your opinion, what are some qualities of a good family planning counselor? What about a not-so-good family planning counselor?
16. How are these skills different, if at all, for counseling women alone, versus counseling women and men together as a couple?
17. How has being a counselor / facilitator / supervisor of couples counseling activities impacted your life, if at all? Professionally and/or personally? Probe:
    - → Has your role in the community changed, or stayed the same, because of your work with the iNGO? Can you give me an example?
    - → Did you learn anything about your own skills or abilities while you were working on the couples counseling activities? Is there something you discovered about yourself?
